# Global metabolic profiling to model biological processes of aging in twins

**DOI:** 10.1111/acel.13073

**Published:** 2019-11-19

**Authors:** Bryan J. Bunning, Kévin Contrepois, Brittany Lee‐McMullen, Gopal Krishna R. Dhondalay, Wenming Zhang, Dana Tupa, Olivia Raeber, Manisha Desai, Kari C. Nadeau, Michael P. Snyder, Sandra Andorf

**Affiliations:** ^1^ Department of Medicine Sean N. Parker Center for Allergy and Asthma Research Stanford University School of Medicine Stanford California; ^2^ Department of Genetics Stanford University School of Medicine Stanford California; ^3^ Quantitative Science Unit Department of Medicine Stanford University School of Medicine Stanford California

**Keywords:** aging, LC‐MS, machine learning, metabolomics, random forest, twins

## Abstract

Aging is intimately linked to system‐wide metabolic changes that can be captured in blood. Understanding biological processes of aging in humans could help maintain a healthy aging trajectory and promote longevity. We performed untargeted plasma metabolomics quantifying 770 metabolites on a cross‐sectional cohort of 268 healthy individuals including 125 twin pairs covering human lifespan (from 6 months to 82 years). Unsupervised clustering of metabolic profiles revealed 6 main aging trajectories throughout life that were associated with key metabolic pathways such as progestin steroids, xanthine metabolism, and long‐chain fatty acids. A random forest (RF) model was successful to predict age in adult subjects (≥16 years) using 52 metabolites (*R* ^2^ = .97). Another RF model selected 54 metabolites to classify pediatric and adult participants (out‐of‐bag error = 8.58%). These RF models in combination with correlation network analysis were used to explore biological processes of healthy aging. The models highlighted established metabolites, like steroids, amino acids, and free fatty acids as well as novel metabolites and pathways. Finally, we show that metabolic profiles of twins become more dissimilar with age which provides insights into nongenetic age‐related variability in metabolic profiles in response to environmental exposure.

## Graphical abstract

Global profiling of plasma metabolites in a cohort of twins by liquid chromatography coupled with mass spectrometry and investigation of the biological processes involved in healthy aging. 

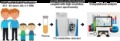

## 1 INTRODUCTION

Aging is a complex and multi‐factorial process that involves system‐wide dysregulation across many biological pathways and molecule types (Hoffman, Lyu, Pletcher, & Promislow, 2017). Understanding mechanisms of healthy aging in humans is important because early detection of deviations from a healthy aging trajectory could be used to promote longevity by delaying, avoiding, or preventing the development of age‐related diseases (Alpert et al., 2019). However, this task has been challenging in part because of the complex interplay of genetic, environmental, and lifestyle factors involved in aging. Metabolomics—the unbiased profiling of a large panel of metabolites—has become the approach of choice to study aging with the realization that metabolic alterations are central to the aging process (Barzilai, Huffman, Muzumdar, & Bartke, 2012; Collino et al., 2013; López‐Otín, Blasco, Partridge, Serrano, & Kroemer, 2013; Montoliu et al., 2014). In addition, metabolomics captures genetic and nongenetic factors since metabolites are influenced by the biology of the host, gut microbial activity, and environmental exposure (Rinschen, Ivanisevic, Giera, & Siuzdak, 2019). Recent studies combining genetic and metabolic profiles in twins were instrumental in quantifying the relative influence of genetic and environmental factors on blood metabolite levels and showed a wide range of heritability largely dependent on chemical classes (Darst, Koscik, Hogan, Johnson, & Engelman, 2019; Kettunen et al., 2012; Long et al., 2017; Shin et al., 2014).

In the recent years, metabolomics has been successfully applied to the study of human aging highlighting many biomarkers and biological pathways associated with age (Chaleckis, Murakami, Takada, Kondoh, & Yanagida, 2016; Menni et al., 2013; Rist et al., 2017; Yu et al., 2012). Through various modeling and experimental methodologies, steroids, amino and fatty acids, and biomarkers of kidney function have been found to have significant associations with aging. However, these studies were often limited by the age range of participants focusing on adults (>18 years old) and by the range of metabolites covered (~100–500).

In this context, we profiled a wide array of 770 metabolites in blood from a cross‐sectional cohort of healthy individuals enriched for twin pairs and aged 6 months to 82 years old. The aim of the study was threefold: (a) describe longitudinal trends of changes with age, (b) identify metabolites and pathways associated with age using random forest (RF) machine learning algorithm (regression and classification models), and (c) investigate biological variability of metabolic profiles in twin pairs with age.

## 2 RESULTS

### 2.1 Cohort characteristics and generation of untargeted metabolomics data

**Figure 1:**
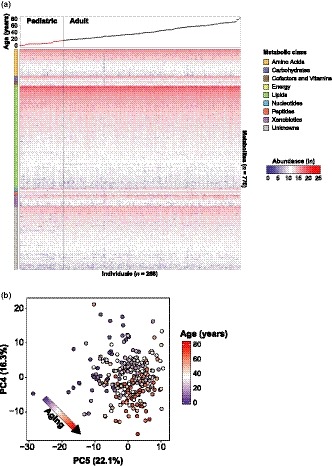
Untargeted metabolomics of aging plasma. (a) Natural log metabolite abundances of all 770 detected metabolites (rows) in 268 individuals (columns) ranging from low (blue) to high (red). Metabolites are ordered by metabolic class and median intensity across all the samples in the study, and individuals are ordered chronologically by age. (b) Principal component analysis using the top two principal components associated with age (*i.e.,* PC4 and PC5). Association with age is calculated via linear modeling of each PC

A cohort consisting of 268 healthy individuals including 125 twin pairs was recruited with the objective of having an even distribution of ages across a human lifespan (6 months to 82 years old) (Figure 1a). Demographic characteristics are shown in Table 1. The cohort was divided in two groups including pediatric (<16 years old) and adult participants (16–82 years) with the assumption that puberty is over at age 16. The pediatric population contained fewer female participants (45.3% vs. 69.8%) and a lower percentage of monozygotic twins among the twin pairs (64% vs. 91%) than the adult population.

**Table 1:** Cohort demographics

	
	<16 years	≥16 years	Total
Individuals (twin sets)	53 (25)	215^a^ (100)	268 (125)
Age in years (mean, *SD*)	7.0 ± 4.1	42.5 ± 17.3	35.5 ± 21.1
Sex % female (*n*)	45.3% (24)	69.8% (150)	64.9% (174)
BMI (mean, *SD*)	16.0 ± 2.7	27.0 ± 6.3	24.9 ± 7.2
Monozygotic individuals % of twins (*n*)	64.0% (32)^b^	91.0% (182)	85.6% (214)

a 12 Singletons, 1 set of triplets.

b Zygosity unknown for 1 twin pair.

Untargeted metabolomics was performed on randomized plasma samples using a broad range liquid chromatography‐mass spectrometry (LC‐MS) platform (Contrepois, Jiang, & Snyder, 2015). This platform uses complementary hydrophilic liquid chromatography (HILIC) and reverse‐phase liquid chromatography (RPLC) separations coupled with high‐resolution mass spectrometry (HRMS). Over 17,000 MS peaks were robustly detected. After filtering and curation, 770 metabolites could be identified (Figure 1a). The named metabolites were categorized in eight main chemical classes and 59 pathways to help biological interpretation and enrichment analysis (Table S1). Annotation confidence levels for each metabolite were provided following the Metabolomics Standards Initiative (MSI) confidence scheme (Table S1) (Schymanski et al., 2014; Sumner et al., 2007).

### 2.2 Metabolic profiles are influenced by age

Variance decomposition was performed on recorded confounders including age, body mass index (BMI), sex, asthma status (GINA score) (Bousquet, 2000), smoking and second‐hand smoking status (Figure S1). Among the tested variables, age showed the most variance in our dataset (1.01% median, 44.50% maximum). In addition, a global aging trajectory in the cohort could be visualized in two dimensions by plotting the principal components (PCs) PC4 (*p* = 2.2E‐12) and PC5 (*p* = 1.4E‐15) that associated the most strongly with age (see methods, Figure 1b).

### 2.3 Metabolic aging trajectories

**Figure 2:**
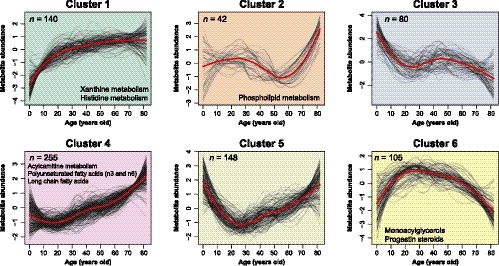
Metabolic aging trajectories. Fuzzy c‐mean clustering of all 770 metabolite abundances fitted to a loess curve and *Z*‐score scaled (black lines), adjusted for sex and BMI, as a function of age in years. Average trend of clusters is shown as a red line. Pathways with FDR < 0.1 are considered significant and displayed

We took advantage of the even age distribution of our cohort across a pediatric and an adult population and defined six clusters based on metabolite aging trajectories (Figure 2, Table S1). Pathway enrichment analysis via hypergeometric testing was used to highlight biological functions enriched in each cluster (FDR < 0.1). Clusters 1, 4, 5, and 6 contained 84% of the metabolites. Metabolites in cluster 1 increased strongly until adult age and then tended to slowly increase with age. The metabolites belonging to this cluster were enriched for xanthine and histidine metabolism. Cluster 4 was enriched for acylcarnitines, as well as long and polyunsaturated fatty acids. Molecules in this cluster tended to remain constant at young age and linearly increased with age after the onset of adulthood. Clusters 5 and 6 presented opposing parabolic shapes with metabolites decreasing at early age and then increasing at adult age for cluster 5 and vice versa in cluster 6. The latter cluster was enriched for monoacylglycerols and progestin steroids. On average, metabolites in cluster 5 reached a minimum at age 29.6 ± 9.2 years (mean, standard deviation) and metabolites in cluster 6 were the most concentrated at age 31.0 ± 11.2 years. These cluster classifications were used to guide biological interpretations in the sections below.

### 2.4 Machine learning reveals metabolites associated with age in a healthy adult population

**Figure 3:**
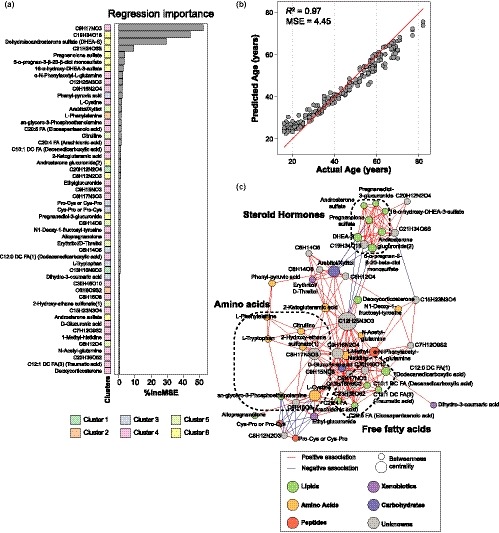
Regression model Random Forest analysis. (a) Significant metabolites in the regression RF model ordered by importance and color‐coded by cluster. (b) 2D scatter plot representing predicted age vs. actual age. *MSE* = mean squared error. (c) Correlation network analysis of significant metabolites in the model. Nodes are colored by metabolic class and sized by betweenness centrality. Edges are colored by association direction

A regression random forest (RF) model analysis to predict age was performed on adults aged 16 years and older to focus on healthy aging in adults (*n* = 215). The dataset was modeled to correct for BMI and sex as these demographics are known to cause variability in metabolic profiles (Darst et al., 2019; Piening et al., 2018) (Figure S1). A model containing 52 metabolites (Figure 3a) successfully predicted healthy aging in adults with a mean squared error of 4.45, R‐square of 0.97, and 54.78% of variance explained (Figure 3b). Interestingly, metabolites in clusters 4 (increase with age) and 6 (decrease with age) dominated representing 50.0% and 19.2% of the selected metabolites, respectively. As expected, androsterone and progesterone derivatives contributed the most to the model (Darst et al., 2019; Feldman et al., 2002) including dehydroisoandrosterone sulfate (DHEA‐S), pregnanolone sulfate, and 16‐α‐hydroxy‐DHEA‐3‐sulfate, of which all presided in cluster 6. In addition to steroid hormones, metabolites involved in amino acid (*i.e.,* phenylacetylglutamine, cystine, phenylalanine, citrulline, tryptophan) and lipid metabolism (*i.e.,* sn‐glycero‐3‐phosphoethanolamine and polyunsaturated fatty acids eicosapentaenoic acid and arachidonic acid) contributed to the RF model. Three unknown metabolites with elemental compositions C9H17NO3, C19H34O15, and C21H34O6S were among the most important metabolites in the model but could not be confidently annotated. Further work would be necessary to formally identify these molecules. Interestingly, C19H34O15 and C21H34O6S resided in cluster 6 and had the same longitudinal pattern of change with age as androsterone and progesterone derivatives suggesting similar function and/or structure (Figure S2A). The unknown metabolite with the elemental composition C9H17NO3 displayed a different aging trajectory with a positive linear association in adulthood as seen in cluster 4.

Correlation network analysis of metabolites included in the RF model revealed distinct functional clusters of co‐regulated metabolites including androgenic and progestin hormones, fatty acids, and amino acids (Figure 3c). Betweenness centrality that represents the importance of the node in the network was calculated for each metabolite. Interestingly, the unknown metabolite with elemental composition C12H25N3O3, residing in cluster 4, was central in the network and was ranked 9th for its importance in the RF regression model, suggesting it may have a critical function in the aging process.

### 2.5 Machine learning reveals metabolites that distinguish pediatric and adult participants

**Figure 4:**
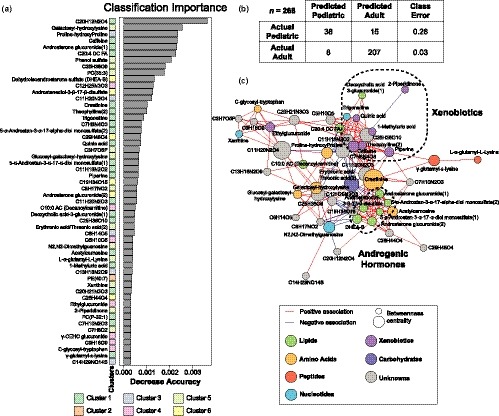
Classification model Random Forest analysis. (a) Significant metabolites in the classification RF model ordered by importance and color‐coded by cluster. (b) Two‐way table of the classification model. (c) Correlation network analysis of significant metabolites in the model. Nodes are colored by metabolic class and sized by betweenness centrality. Edges are colored by association direction

Next, we were interested in identifying the metabolites that would classify samples from pediatric (*n* = 53) and adult (*n* = 215) participants. An RF classification model determined 54 metabolites (Figure 4a) important in differentiating pediatric participants from adults with an out‐of‐bag estimate of error of 8.58%. In contrast to the regression model, the classification model selected metabolites enriched in cluster 1 (more abundant in adults) and 5 (less abundant in adults) representing 40.7% and 24.1%, respectively. The classification error was very small for adults (3%) and was greater for pediatric participants (28%) likely because of the smaller sample size of the latter (Figure 4b). As expected, metabolite biomarkers for the consumption of coffee (*i.e.,* caffeine, theophylline, trigonelline, quinic acid and 1‐methyl uric acid), residing in cluster 1, as well as androsterone derivatives (*i.e.,* androsterone glucuronide and DHEA‐S) in cluster 6, were important in the RF classification model. All these molecules were more abundant in adult participants. In addition, we observed two glycosylated amino acids that were important to the model, namely galactosyl‐hydroxylysine and glucosyl‐galactosyl‐hydroxylysine. The level of these molecules was decreased in the adult group (Figure S2B). We also found creatinine, C‐glycosyl tryptophan, γ‐carboxyethyl hydroxychroman (CEHC) glucuronide, γ‐glutamyl‐ε‐lysine, α‐glutamyl‐lysine, proline‐hydroxyproline and acetylcarnosine as main contributors in the model.

Correlation network analysis of metabolites selected in the model highlighted a cluster of steroid hormones containing androsterone derivatives as well as a cluster of xenobiotics coming from coffee consumption (Figure 4c). Interestingly, creatinine had a central position in the network and positively associated with 20 metabolites (Bonferroni adjusted *p*‐value <.01) bridging the two main clusters containing androgenic hormones and xenobiotics. Clinically, plasma creatinine is used to monitor renal function and was found to increase with age in our cohort (Shlipak et al., 2009; Tiao, Semmens, Masarei, & Lawrence‐Brown, 2002).

Interestingly, the metabolites selected in the two RF models generated to predict age in a healthy adult population and to classify pediatric and adult participants presented little overlap (*n* = 7 metabolites). While the regression RF model successfully selected metabolites involved in energy metabolism (*i.e.,* amino acid and lipid metabolism), the classification model selected many xenobiotics including metabolites derived from the consumption of coffee.

### 2.6 Variability of metabolic profiles in twin pairs as a function of age

**Figure 5:**
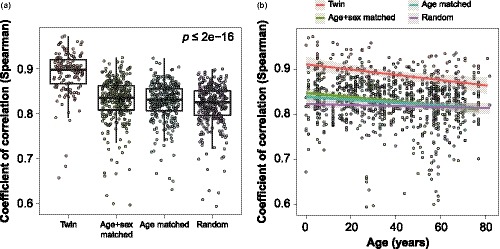
Variability of metabolic profiles in twins. (a) Spearman's correlation of the whole collection of metabolites between various pairs of individuals. *p*‐value shown was calculated via Kruskal–Wallis test. (b) Spearman's correlation plotted as a function of age shows a significant decreasing trend over time in twins

Since our cohort was enriched for twin pairs, and specifically monozygotic pairs, we assessed the variability of metabolic profiles between twins with age. As expected, we found that the Spearman rank correlation coefficient calculated across all detected metabolites was significantly higher between twin samples (median rho = 0.894) than between age‐ and sex‐matched participants (median rho = 0.835, *p* = 2.7E‐31), age‐matched participants (median rho = 0.831, *p* = 6.4E‐36), and randomly selected pairs (median rho = 0.825, *p* = 8.5E‐40) (Figure 5a). Linear modeling of pairwise Spearman's correlation as a function of age resulted in a significant negative term for age in twins (*p* = 1.1E‐3) as well as age‐ and sex‐matched (*p* = 4.1E‐4) and age‐matched pairs (*p* = 5.2E‐3). Random pairs without age or sex matching showed no significant association of Spearman's rank correlations and age (*p* = .36) (Figure 5b). The results in twins were not driven by twin status since the cohort had more dizygotic twins in pediatric than in adult individuals (Table 1). Via linear model, we calculated that the pairwise correlation between metabolic profiles of a given twin pair decreases by 0.0005906 each year. These observations may reflect the impact of differential environmental exposure on twin pairs that accumulate with age.

## 3 DISCUSSION

Metabolomics has become a popular approach to study biological processes associated with age and has been applied in various human studies (Barzilai et al., 2012; Collino et al., 2013; López‐Otín et al., 2013; Montoliu et al., 2014; Rinschen et al., 2019). Our study expands on previous work by profiling 770 chemically diverse plasma metabolites from twin pairs of pediatric and adult participants (*n* = 268) with an even distribution of age (6 months to 82 years old). We used a sensitive and robust LC‐MS platform involving complementary hydrophilic liquid chromatography (HILIC) and reverse‐phase liquid chromatography (RPLC) separations to capture a wide array of metabolites covering 59 biochemical pathways (Contrepois et al., 2015). Our novel bioinformatic approach combining unsupervised clustering, machine learning and correlation networks recapitulated known biology but also led to discoveries.

Regression and classification RF selected a small number of metabolites that successfully predicted age in an adult population and classified pediatric and adult participants. Xenobiotics were among the strongest predictors that differentiated pediatric and adult participants and included many metabolites associated with coffee consumption (*i.e.,* caffeine). These molecules were much more abundant in the blood of adult subjects (cluster 1) which was expected given that coffee is frequently ingested by adults but rarely by children (Azam, Hadi, Khan, & Hadi, 2003; Fulgoni, Keast, & Lieberman, 2015). These results validate our study demonstrating that we generated good quality untargeted metabolomics data and we employed an efficient bioinformatic approach to extract meaningful biology from the data.

The classification model revealed two glycosylated forms of hydroxylysine found in collagen. Galactosyl‐hydroxylysine is a marker of bone resorption (Al‐Dehaimi, Blumsohn, & Eastell, 1999). Previous studies found galactosyl‐hydroxylysine to be (a) higher in the blood of girls compared with premenopausal women reflecting high bone turnover and (b) higher in postmenopausal compared with premenopausal women reflecting higher bone resorption rates in postmenopausal older women due to estrogen deficiency related with menopause (Feng & McDonald, 2011). Our data validate these findings with galactosyl‐hydroxylysine describing a positive parabolic trajectory (cluster 5), and we found that glucosyl‐galactosyl‐hydroxylysine follows the same aging trajectory.

As expected, androsterone and progesterone derivatives were selected by machine learning algorithms because they vary greatly with age (Darst et al., 2019; Feldman et al., 2002). Dehydroepiandrosterone sulfate (DHEA‐S), a sulfate ester of adrenal steroid dehydroepiandrosterone (DHEA), was selected with both models. Its levels are known to correlate strongly with age, and it has been shown to be an important regulator of some age‐related biological processes (Feldman et al., 2002). However, neither DHEA nor low‐dose testosterone replacement in elderly people presented physiologically relevant beneficial effects (Nair et al., 2006). Steroid hormones showed a negative parabolic pattern, with levels spiking at around age 30 that decay throughout adulthood (cluster 6).

In addition to steroid hormones, many amino acids and lipids were selected in the models. We detected some expected changes including an accumulation with age of citrulline and cystine, a dimeric form of cysteine, presumably due to impaired muscle function in the elderly (Pitkänen, Oja, Kemppainen, Seppä, & Mero, 2003). Importantly, our study included pediatrics samples which allows to discriminate metabolites based on their changes before puberty. Even though citrulline and cystine are both known to increase with age, we found that they belonged to different clusters. Citrulline, in cluster 5, presented a parabolic curve with decreased levels with age before puberty followed by an increase with age in adults while cystine in cluster 4 remained constant before puberty and increased with age in the adult population. These findings demonstrate the power of our approach to discover new metabolic aging trajectories throughout the human lifespan.

We also observed some modified amino acids that accumulated with age (cluster 4). Phenylacteylglutamine was important in the regression model (8th position) and has been described as a marker of healthy aging (Collino et al., 2013). Interestingly, it is produced by the gut microbiome from amino acid fermentation which is in line with the growing body of literature related to gut microbial composition shift with age (Nagpal et al., 2018; Yatsunenko et al., 2012). We also detected C‐glycosyl tryptophan in cluster 4 that was shown to strongly correlate with chronological age (Menni et al., 2013).

Two polyunsaturated fatty acids, arachidonic acid (AA), and eicosapentaenoic acid (EPA) were highlighted in our analysis and positively associated with age (cluster 4). Even though docosahexaenoic acid (DHA) was not selected in the models, it presented the same longitudinal trajectory as AA and EPA (Otsuka, Kato, Imai, Ando, & Shimokata, 2013). These molecules are important modulators of cardiovascular health and inflammation (James, Gibson, & Cleland, 2000). In a recent study, Deelen et al. (2019) identified a metabolic signature containing 14 biomarkers predictive of all‐cause mortality. Interestingly, polyunsaturated fatty acids were important molecules both in their model to predict all‐cause mortality independent of age and sex and in our model to predict age.

Correlation network analysis revealed that creatinine presented the most connections in the classification model highlighting its central role in the aging process. Creatinine is an important molecule that is used to measure renal function in the clinic and was found to increase strongly with age in the pediatrics cohort (cluster 1). Unknown metabolite C12H25N3O3 was central in the correlation network for the regression model bridging steroid hormones, free fatty acids, and amino acids which highlights its potential functions in the biological process of aging in healthy adults. Interestingly, some unknown metabolites were among the most important molecules in our models with C9H17NO3 that was ranked 1st in the regression RF model.

Importantly, the longitudinal trajectories of metabolites with age described in our analysis are best generalized as parabolic. Hence, a model using a second‐order term (*y* = β*age^2^ + β*age) should be more appropriate than a simple linear model of age (*y* = β*age) to identify metabolites associated with age. Linear models have been widely used potentially impacting accuracy and significance of the findings (Menni et al., 2013; Yu et al., 2012).

We are the first, to our knowledge, to show a decrease in Spearman's rank correlation between twins’ untargeted metabolic profiles as age increases. This observation is likely explained by the influence of nongenetic factors including lifestyle and environmental exposure on the metabolome. This increased variability with age was also observed in other biological systems such as the immune system (Brodin et al., 2015; Cheung et al., 2018). The average change in correlation over our cohorts age range of 80 years between twins (−0.0005906/year * 80 years = −0.046) is on the order of the difference between the average of the twin correlations and the average of the random age‐matched correlations over the same age range (−0.073). Comparing these two values helps to contextualize changes in correlations due to genetic factors, or due to aging. The relatively high correlations of metabolic profiles among randomly age‐matched participants reflect the small variability in abundance of most metabolites between healthy individuals. As an example, a modest increase of circulating branched‐chain amino acids (10%–20%) most consistently differentiates individuals with insulin resistance and/or type 2 diabetes (Guasch‐Ferré et al., 2016; Schüssler‐Fiorenza Rose et al., 2019).

This study should be assessed in the context of its limitations. First, the participants were not required to fast before the blood draw and blood samples were collected at any time of the day. However, we demonstrated the biological relevance of the metabolomics data generated since we were able to reproduce main expected changes associated with age. Second, our study is cross‐sectional by design which limits its statistical power due to inter‐individual variability of metabolic profiles. To circumvent this issue, two recent studies profiled metabolites in a longitudinal fashion (Chak et al., 2019; Darst et al., 2019). Even though these studies improve the robustness of the findings, they do not give insights into mechanisms resulting in the observed metabolic changes. Longitudinal multi‐omics studies will likely be key to unravel molecular mechanisms that results in age‐dependent metabolism dysregulation in human plasma by integrating information from multiple regulatory levels (Piening et al., 2018; Zhou et al., 2019). Lastly, younger twins most likely still live together or have lived apart for a shorter period of time and, thus, have more recently shared environmental exposures than older twins, which has to be considered when interpreting the results of the twin analysis.

Overall, we show that our computational approach involving clustering, RF machine learning, and network analysis was successful to describe metabolic aging trajectories throughout life and discover metabolites and pathways dysregulated with age. Additionally, we revealed an increased variability of metabolic profiles in twin pairs with age suggesting accumulating effects of differential exposure on human metabolome.

## 4 EXPERIMENTAL PROCEDURES

### 4.1 Study design and participants

A cohort of 268 participants enriched for twins was recruited as part of an observational study (ClinicalTrials.gov Identifier: NCT01613885), which included twins of all ages from sites in Washington and California between 2010 and 2016. Individuals with an active infection, history of cancer, autoimmune disease, or that use oral steroids or immunomodulators were not included in this analysis. As such, participants were considered healthy. The cohort was composed of 125 twin pairs, 1 triplet set, and 15 singlets consisting of family members or instances of only one twin arriving at the appointment. In addition to a routine examination, participants completed a detailed questionnaire regarding their medical history as well as dietary habits. Individual‐level demographic information, self‐reported disease status, and self‐reported frequent medication usage are provided in Table S2. This study was conducted with the approval of Stanford University Institutional Review Board (IRB‐19495), and all subjects provided signed informed consent before participation.

### 4.2 Blood collection and plasma preparation

Intravenous whole‐blood was collected nonfasted and at any time of the day in 10 ml EDTA‐vacutainer tubes, and plasma was prepared within 24 hr following centrifugation at 2,200 rpm for 20 min at 24°C. Blood was kept at room temperature prior to processing. The top layer plasma was pipetted off, aliquoted, and immediately frozen at −80°C. Plasma aliquots were de‐identified in agreement with CFR/GCP (code of federal regulation/good clinical practice) guidelines. Samples from twin pairs were collected the same day at the same time.

### 4.3 Chemicals

LC‐MS‐grade solvents and mobile phase modifiers were obtained from Fisher Scientific (water, acetonitrile, methanol, formic acid) and Sigma‐Aldrich (ammonium acetate).

### 4.4 Metabolite extraction

Fresh plasma aliquots were used for the study, and metabolites were extracted in a randomized order as previously described (Contrepois et al., 2015). Briefly, metabolites were prepared from 100 µl of plasma using 1:1:1 acetone:acetonitrile:methanol, evaporated to dryness under nitrogen, and reconstituted in 1:1 methanol:water.

### 4.5 Untargeted metabolomics profiling

Metabolic extracts were analyzed four times using hydrophilic liquid chromatography (HILIC) and reverse‐phase liquid chromatography (RPLC) separation in both positive and negative ionization modes. Data were acquired on a Thermo Q Exactive plus mass spectrometer equipped with a HESI‐II probe and operated in full MS scan mode. MS/MS data were acquired on pool samples consisting of an equimolar mixture of 100 randomized samples in the study. HILIC experiments were performed using a ZIC‐HILIC column 2.1 × 100 mm, 3.5 μm, 200Å (Merck Millipore), and mobile phase solvents consisting of 10 mM ammonium acetate in 50/50 acetonitrile/water (A) and 10 mM ammonium acetate in 95/5 acetonitrile/water (B). RPLC experiments were performed using a Zorbax SBaq column 2.1 × 50 mm, 1.7 μm, 100Å (Agilent Technologies), and mobile phase solvents consisting of 0.06% acetic acid in water (A) and 0.06% acetic acid in methanol (B).

Data quality was ensured by (a) sample randomization for metabolite extraction and data acquisition, (b) multiple injections of a pool sample to equilibrate the LC‐MS system prior to run the sequence (12 and 6 injections for HILIC and RPLC methods, respectively), (c) spike‐in 9‐labeled internal standards (IS) during the sample preparation to control for extraction efficiency and evaluate LC‐MS performance, (d) checking mass accuracy, retention time, and peak shape of IS in every samples, and (e) injection of a pool sample every 10 injections to control for signal deviation with time.

### 4.6 Data processing and metabolite annotation

Data from each mode were independently analyzed using Progenesis QI software v2.3 (Nonlinear Dynamics). Metabolic features from blanks and that did not show sufficient linearity upon dilution were discarded (*R* ^2^ < .6). Only metabolic features present in >33% of the samples were kept for further analysis, and missing values were imputed using minimum value imputation (Tyanova et al., 2016). MS signal drift with time was corrected for each metabolite using LOESS normalization method (locally estimated scatterplot smoothing Local Regression) on pool samples. Data from each mode were merged, and metabolites were formally identified by matching the fragmentation spectra and retention time to analytical‐grade standards when possible or matching experimental MS/MS to fragmentation spectra in publicly available databases. We used the Metabolomics Standards Initiative (MSI) level of confidence to grade metabolite annotation confidence (level 1 ‐ level 4) (Table S1). Level 1 represents formal identifications where the biological signal matches accurate mass, retention time, and fragmentation spectra of an authentic standard ran on the same platform. For level 2 identification, the biological signal matches accurate mass and fragmentation spectra available in METLIN database. Acylcarnitines, free fatty acids, and complex lipids do not necessarily all have MS/MS data in public databases but were annotated based on expected signature fragments. Level 3 represents putative identifications that are the most likely name based on previous knowledge of blood composition. Level 4 consists in unknown metabolites. After careful annotation of the metabolic features, a total of 770 metabolites were measured using our metabolite profiling platform. Metabolites were then categorized in classes and pathways based on the KEGG database where possible. Metabolite abundances (spectral counts) for all participants can be found in Table S3.

### 4.7 Data analysis and visualization

#### 4.7.1 Heatmap and principal component analysis

Natural log metabolite abundances of all 770 detected metabolites (rows) in 268 individuals (columns) were plotted as a heatmap using the R package “pheatmap” (v1.0.8). Principal component analysis was performed using the function prcomp in the base “stats” package in R (v3.3.3). Abundances were natural log‐transformed and scaled by metabolite using the *Z*‐score method (mean of zero and standard deviation of 1) prior to the analysis. In order to identify the 2 principal components (PCs) most strongly associated with age, we applied linear regression models between PC scores and age for each PC and selected the 2 PC with the smallest *p* values. The 4th and 5th PCs ordered by overall variation were selected.

#### 4.7.2 Variance partition analysis

Variance partition was performed using the “variancePartition” package in R (v1.4.2). Age and BMI were considered continuous variables whereas sex, asthma status (GINA scoring) defined by a pulmonologist (Bousquet, 2000), smoking, and second‐hand smoking exposure were considered categorical variables.

#### 4.7.3 Clustering of metabolic aging trajectories

Fuzzy c‐means clustering was performed using the R package “Mfuzz” (v2.20.0). Data inputted into the clustering algorithm were created by fitting a LOESS model of age in years (6 months to 82 years) vs. metabolite abundances using span = 0.75 with R package “stats” (v3.3.3). Data were then scaled so that the mean equal 0 and the standard deviation equal 1 for each metabolite. We calculated the minimum centroid distance for a range of cluster numbers and chose six clusters as the optimal number of clusters.

#### 4.7.4 Random forest prediction modeling

Two predictive models were generated using random forest (RF) algorithm (“RandomForest” package in R (v4.6)). The regression model used age as a continuous variable in adult population (≥16 years old). Importance values were assessed (“importance = T”). The classification model used age as a discrete variable to distinguish adults (≥16 years old) from pediatrics subjects (<16 years old). Abundance profiles of all metabolite were natural log‐transformed and adjusted for sex and BMI using a linear model prior to modeling. Significance was defined as the model importance metric greater than the absolute value of the most negative metabolite. Sub‐sampling (“sampsize” parameter) was utilized when performing the classification model on the adult group to account for the unbalanced size between pediatric and adult populations in the cohort.

#### 4.7.5 Correlation network analysis

Pairwise Spearman's rank correlations were calculated using the R package “Hmisc” (v3.15–0), and weighted, undirected networks were plotted with “igraph” (v0.7.1). Correlations with Bonferroni adjusted *p*‐values below 0.01 were included and displayed via the Fruchterman–Reingold method. Only vertices with at least one connection were plotted. Nodes were color‐coded by chemical classes, and their size represents the betweenness centrality.

#### 4.7.6 Pathway enrichment analysis

Pathway enrichment analysis was performed using hypergeometric tests and the background of the detected metabolites in the study. *p* values were adjusted for multiple comparisons using Benjamini and Hochberg correction (Benjamini, 2010).

#### 4.7.7 Twin metabolic variability

To evaluate metabolic variability between twins, we calculated Spearman's rank correlations using all the detected metabolites (R package “stats” (v 3.3.3)). We also calculated the Spearman's rank correlations between randomly selected, age‐matched, and between randomly selected, sex‐ and age‐matched pairs. Age matching was done by randomly selecting an individual within three years of age. We also included a set of randomly selected pairs without any matching restrictions. Singlets were not included in this analysis. For triplet participants, we randomly picked two sex‐matched triplets.

## CONFLICT OF INTEREST

MPS is a cofounder of Personalis, SensOmics, January, Filtricine, Qbio, and Akna. KCN has received funding from, is currently funded by, or cofounded the following: the NIH, Food Allergy Research & Education, End Allergies Together, Before Brands, Alladapt Immunotherapeutics, Adare Pharmaceuticals, AstraZeneca, Novartis, Genentech, Astellas, DBV Technologies, ForTra, Aimmune Therapeutics, Regeneron, Nestle, Sanofi, ImmuneWorks, Cour, Allergenis, Ukko, and the Environmental Protection Agency. The remaining authors declare no relevant competing interests.

## AUTHORS’ CONTRIBUTION

KN, MPS, and KC conceived and assisted with the design of the project. BB and KC wrote the manuscript with assistance from SA and KN. KC and BL‐M performed the experiment with assistance from WZ. BB and KC performed data analysis with assistance from SA, GD, and MD. DT, BB, and OR assisted in the clinic.

## Data Availability

The data that support the findings of this study are shared as supplemental tables (Tables S2 and S3).
