# Supplementary material for: Global metabolic profiling to model biological processes of aging in twins
Source: Aging Cell. 2019 Nov 19;19(1):e13073. doi: 10.1111/acel.13073 (PMC6974708; doi:10.1111/acel.13073)
Supplement: Supplementary file 1 [file ACEL-19-e13073-s001.pdf]

Supplementary Figure 1

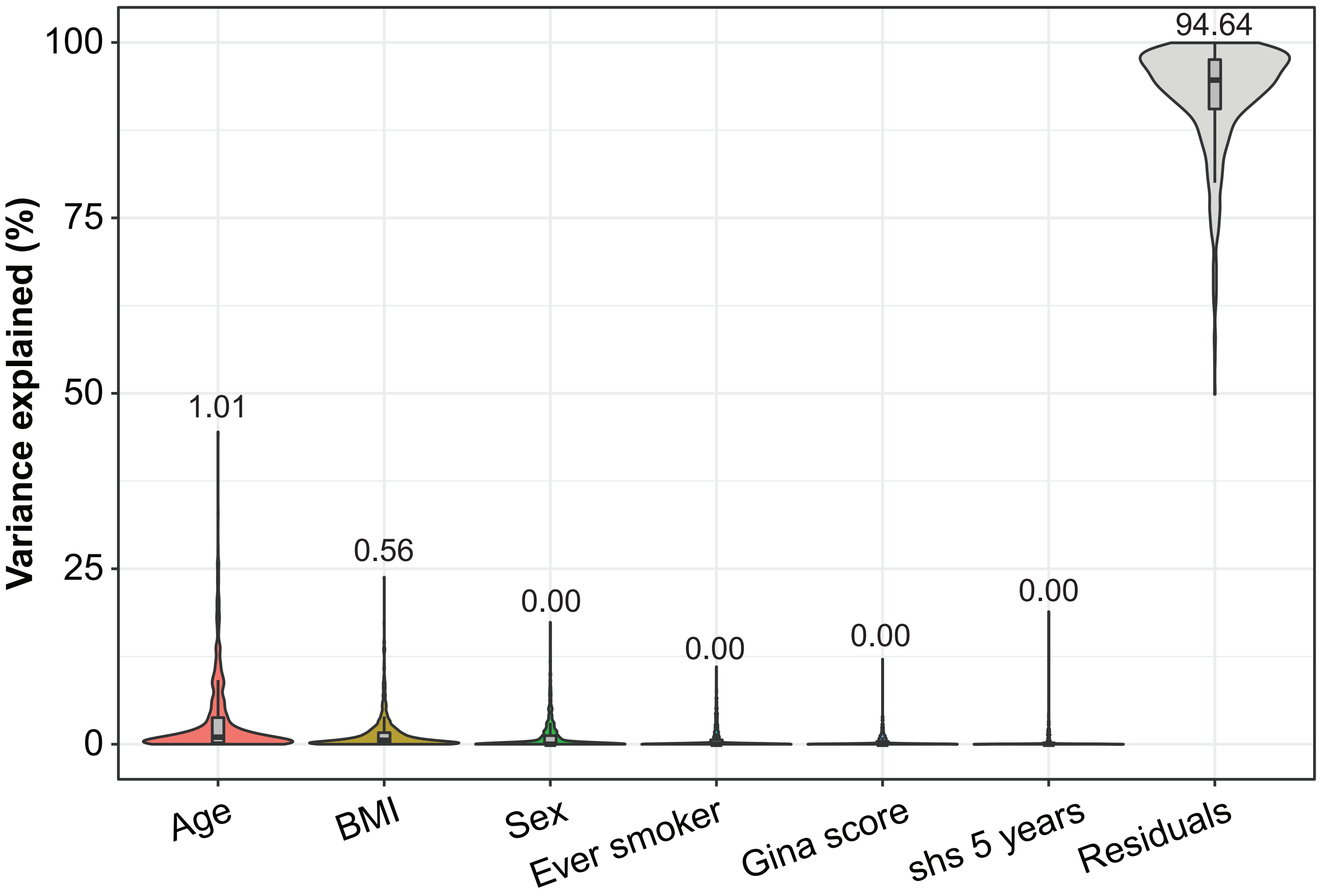

Figure S1. Variance partition of metabolic profiles among various clinical characteristics. The median values are displayed.

Supplementary Figure 2

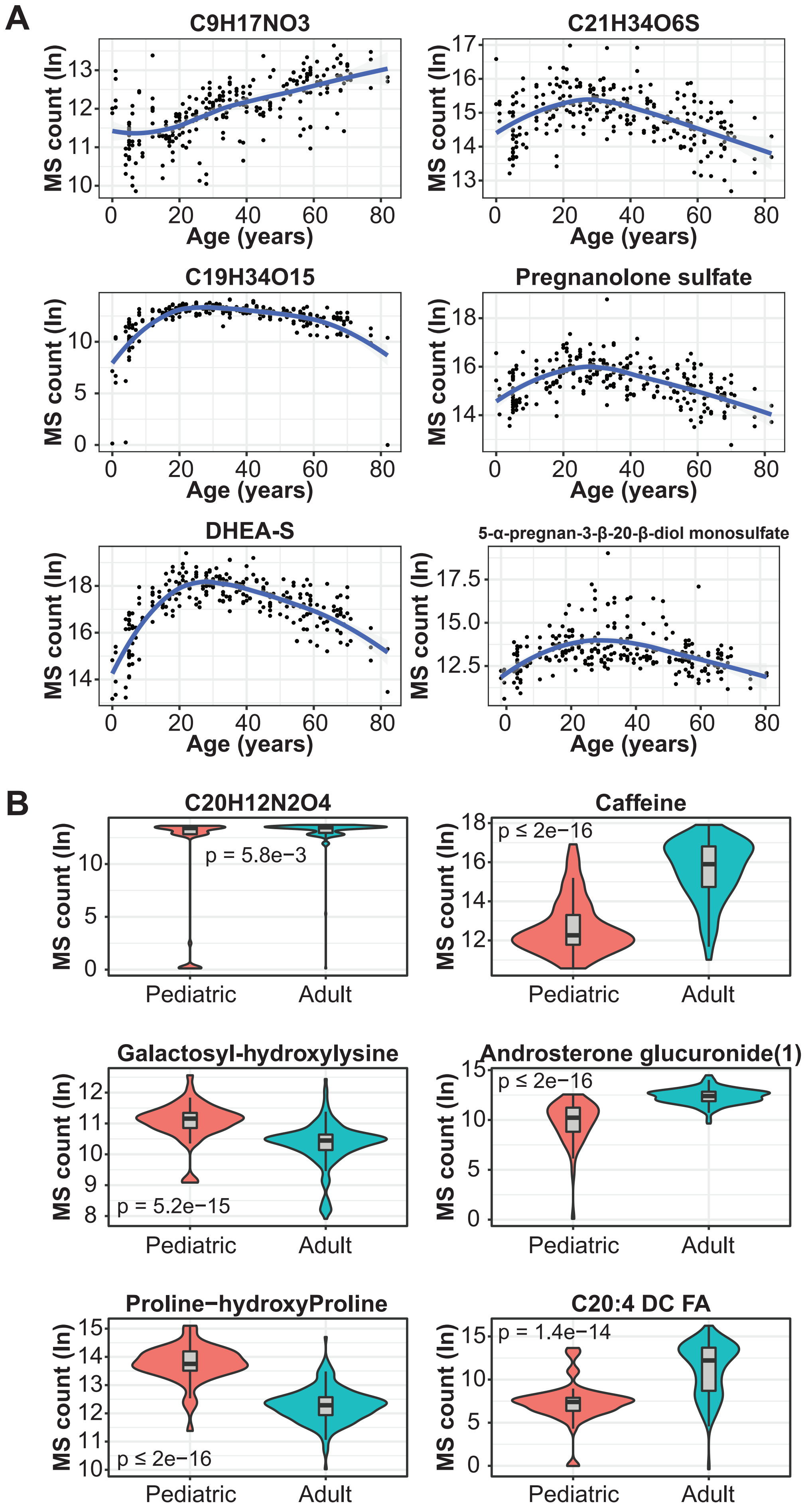

Figure S2. Top 6 metabolites in RF (A) regression and (B) classification models ordered by importance. The blue line fit was calculated via LOESS method. Unadjusted p-values were calculated via Mann-Whitney U test.
